# Effectiveness of peer‐delivered sleep health education and social support in increasing OSA evaluation among at‐risk blacks

**DOI:** 10.1111/jsr.14213

**Published:** 2024-05-21

**Authors:** Girardin Jean‐Louis, P. Jin, R. Moise, J. Blanc, A. Rogers, O. M. Bubu, D. Chung, F. Zizi, A. A. Seixas

**Affiliations:** ^1^ Department of Psychiatry and Behavioral Sciences, Miller School of Medicine University of Miami Miami Florida USA; ^2^ Department of Neurology, Miller School of Medicine University of Miami Miami Florida USA; ^3^ Department of Population Health NYU Grossman School of Medicine New York City New York USA; ^4^ Department of Health and Human Services St John's University Jamaica New York USA; ^5^ Department of Informatics and Health Data Science, Miller School of Medicine University of Miami Miami Florida USA

**Keywords:** apnea, disparity, patient‐centred outcomes, race/ethnicity, sleep health, stakeholder

## Abstract

To assess the effectiveness of culturally and linguistically tailored, peer‐delivered obstructive sleep apnea education and of social support to increase adherence to physician‐recommended obstructive sleep apnea evaluation among blacks. In a two‐arm randomised controlled trial, we ascertained the effectiveness of peer‐delivered obstructive sleep apnea education in increasing obstructive sleep apnea evaluation among 319 blacks at risk of obstructive sleep apnea (intervention = 159 and control = 160); their average age was 47 ± 12.9 years, and 41% were male. Obstructive sleep apnea risk was assessed with the Apnea Risk Evaluation System questionnaire, administered in community venues. Participants in the intervention arm received tailored obstructive sleep apnea education during a 6 month period; those in the control arm received standard sleep and healthy lifestyle information. Analysis focussed on the effectiveness of peer‐delivered obstructive sleep apnea education on adherence to obstructive sleep apnea evaluation, but also considered the role of psychosocial factors. The results showed no significant differences in baseline demographic and clinical measures when contrasting participants in the study arms. The adherence rates for home‐based obstructive sleep apnea evaluation in the intervention and control arms were 45.9% and 45.6%, respectively. Overall, participants in both study arms (adherers) who underwent obstructive sleep apnea evaluations were likely to experience a greater level of social support (8.2 ± 2.4 vs. 7.3 ± 2.4; *p* = 0.06). Moreover, adherers showed greater psychosocial scores (i.e., Dysfunctional Beliefs and Attitudes about Sleep scale, Apnea Beliefs Scale (ABS) (and Apnea Knowledge) compared with non‐adherers (6.0 ± 1.8 vs. 4.9 ± 2.2; *p* = 0.02; 77.0 ± 7.1 vs. 73.2 ± 7.4; *p* = 0.04, and 6.4 ± 3.1 vs. 7.6 ± 2.4; *p* = 0.06, respectively). The results of the present randomised controlled trial favoured a potential role of peer‐based social support and psychosocial factors, associated with obstructive sleep apnea adherence behaviour.

**BRIEF SUMMARY**

**Current knowledge/Study rationale:** Individuals of the black race/ethnicity are disproportionately affected by obstructive sleep apnea (OSA), relative to their white counterparts. Notwithstanding these large OSA disparities, few individualised interventions have specifically aimed at reducing OSA‐related morbidity and mortality in this underserved population.

**Study impact:** This randomised controlled trial assessed the effectiveness of a culturally and linguistically tailored, peer‐delivered OSA education (PEERS‐ED) and of social support to increase adherence to physician‐recommended OSA evaluation among blacks. Future studies should explore whether peer‐delivered social support could be a key ingredient in interventions intended to increase behavioural change leading to increased OSA adherence behaviour among blacks.

## 1 INTRODUCTION

Obstructive sleep apnea (OSA) is a prime example of a cardiovascular‐related disease that can be treated optimally to improve haemodynamic functions and metabolic diseases (Sajkov et al., 1999) and to reduce morbidity (Milleron et al., 2004) and early mortality (Harsch et al., 2005). However, individuals of the black race/ethnicity generally underuse available sleep services, likely due to poor sleep health literacy or dysfunctional beliefs about sleep (Harsch et al., 2005). Our controlled studies performed among blacks at high risk in practice‐based settings revealed that about one‐third adhered to physician‐recommended evaluation (Jean‐Louis et al., 2008; Seixas et al., 2016). This is noteworthy since 59% were at risk for OSA (Jean‐Louis et al., 2008; Seixas et al., 2016). Notably, 90% of blacks undergoing laboratory‐based assessment were diagnosed with OSA (Jean‐Louis et al., 2008; Seixas et al., 2016). These findings alarm sleep epidemiologists and public health advocates alike given the pervasive racial/ethnic disparity in OSA (Mansfield et al., 2004). Consistent with the national mandate to improve health equity among US racial/ethnic groups, implementing novel behavioural sleep models to navigate blacks with OSA symptoms in the healthcare system is a high priority of Healthy People 2030.

Much of the literature addressing racial/ethnic disparities in adherence behaviour toward medical evaluation points to a lack of access to care (Wright et al., 1997). However, evidence suggests that reduced access is largely attributable to a general mistrust of the healthcare system (Cheatham et al., 2008; Jean‐Louis et al., 2017). Individuals from racial/ethnic minority communities do not routinely seek medical care due in part to long‐held attitudes/beliefs and cultural norms that are often incongruous with recommended healthful practices (Jean‐Louis et al., 2017). Notwithstanding these observations, it bears noting that blacks activated in the healthcare system tend to report better satisfaction with care, trust building, and active participation in clinical encounters when providers are of similar racial/ethnic traditions (Perez‐Stable et al., 1997). This lends credence to the notion that innovative behavioural OSA models addressing barriers preventing recommended evaluation practices must integrate a caring and trust‐building approach, anchored by change agents of similar sociodemographic characteristics to maximise the uptake of appropriate health education (Jean‐Louis et al., 2017; Seixas et al., 2016).

In that regard, peer health education has emerged as a successful model integrating a caring and trust‐building approach leveraging credible health champions sanctioned by community elders (Nyswander, 2002). Peer health education is a modality that enables peers to teach or share health information, values, and behaviours with individuals sharing similar demographic and sociocultural characteristics (Satterfield, 2010). It bridges the gap between communities and the healthcare system, promotes wellness by providing culturally and linguistically appropriate health information, assists peers in navigating the healthcare system and advocates for their individual needs, and builds community capacity (Satterfield, 2010). The key element is that the intended audience must trust and respect the peer health educators. Thus, peer educators must in turn be conversant in a specific health area and intimately familiar with the social sanctions, rituals, values, and rules of conduct within the community to ensure intervention goals are achieved (Williams et al., 2016).

Whereas the exposure to peer education increases access to health services among individuals at risk for morbidity and mortality, it may be insufficient to achieve desired adherence goals. Success of behavioural interventions is in part attributed to the investigators’ ability to tailor intervention strategies to address known barriers in the intended community (Cheatham et al., 2008). Peer education is an effective tool to motivate blacks with symptoms of OSA to engage in healthful practices in line with their own level of readiness and efficacy to navigate the healthcare system, but blacks often need social support to reduce decisional conflicts hindering positive change (Green & Kelly, 2004). Evidence shows that social support influences the likelihood that individuals will change/initiate a new behaviour or maintain a new behaviour once initiated (Lewis et al., 2010). Of note, the role of interpersonal influence, a fundamental component of peer health education, has profound effects on anchoring behavioural change (Ogedegbe et al., 2004).

Our Sleep Disparities Workgroup has been successful in ascertaining barriers and facilitators to adequate OSA care in minoritised communities (Jean‐Louis et al., 2017). Direct engagement efforts have led to successful stakeholder‐endorsed interventions to increase adherence to recommended OSA evaluation in practice‐based settings as well as the development of a web‐based sleep health education platform to increase sleep health literacy among blacks (Jean‐Louis et al., 2017; Jean‐Louis et al., 2020). Stakeholders in the present RCT represented community leaders, health champions, and patients with OSA who have guided the process of designing the study, recruitment of credible and representative health educators, and community events at barbershops, beauty salons, and places of worship to solicit and enrol participants in the RCT. We have learned that this approach is very effective in addressing OSA barriers (e.g., poor sleep literacy to make informed decisions, negative attitudes/beliefs about sleep, and lack of social support) (Jean‐Louis et al., 2017; Shaw et al., 2012), but its effectiveness in increasing adherence rates to OSA evaluation among blacks in traditional settings has not been systematically ascertained. The primary aim of the present study was to use a stakeholder‐engaged research model (Pandi‐Perumal et al., 2015) to evaluate the effectiveness of peer‐delivered, tailored OSA health education. The secondary aim was to examine the predictive role of peer‐based social support in increasing the adherence rates to recommended OSA evaluation among blacks at risk of OSA. We also assessed the contribution of several psychosocial factors in delineating OSA adherence behaviour in this unique population.

## 2 METHODS

### 2.1 Participants

This is an NIH‐funded stakeholder‐engaged study enrolling patients from barbershops, beauty salons, and places of worship in New York. We used a randomised controlled trial (RCT) design to assess the effectiveness of a culturally and linguistically tailored peer‐delivered intervention (PEERS‐ED) and of social support in increasing adherence to physician‐recommended evaluation of OSA among blacks. Participants were eligible if their self‐reported race/ethnicity was black/African American, were ≥ 18 years old, and were at risk of OSA (Seixas et al., 2018). They were excluded if they had documented coexisting sleep problems, presence of illness or disability in which death was expected within 2 years of enrolment, non‐English speaking, impaired cognitive or functional ability, were currently being treated for sleep apnea, or had another family member participating in the study.

**FIGURE 1:**
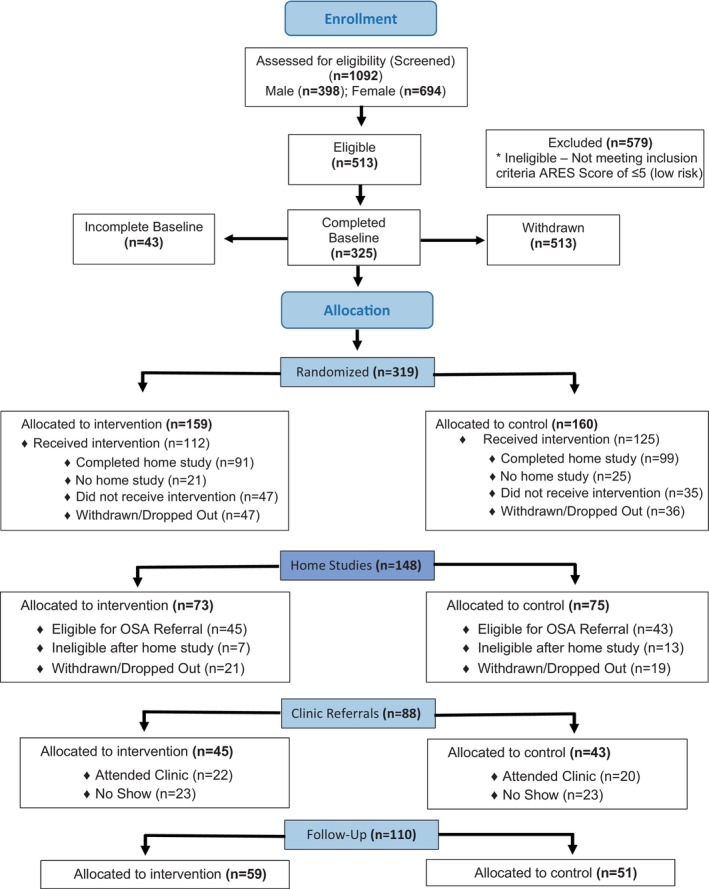
Consort diagram depicting the flow of participants in the peer‐delivered obstructive sleep apnea education trial.

As illustrated in the CONSORT flowchart (Figure 1), a total of 1092 patients were screened for eligibility. Of those, 319 patients were randomised to either the intervention arm (*n* = 159) or the attention control arm (*n* = 160). A total of 83 (26%) patients withdrew from the trial during the 6 month intervention; a total of 110 participants provided follow‐up ascertainment data. All participants provided informed consent under the supervision of the NYU Langone Health institutional review board. The trial was conducted in accordance with the Consolidated Standards of Reporting Trials (CONSORT) Statement on randomised trials for non‐pharmacological treatment and the Standard Protocol Items: Recommendations for Interventional Trials SPIRIT checklist (Zwarenstein et al., 2008).

### 2.2 Procedures

From 2014 to 2019, patients of the black race/ethnicity were enrolled in the study using direct solicitations in venues sanctioned by the Community Steering Committee, including barbershops, beauty salons, places of worship, civic centres, and health fairs in New York City. Research assistants engaged potential participants to solicit their interest; they explained the nature of the study and screened them to determine eligibility. Each participant received $10 for providing complete baseline screening data; those completing both phases of the trial post randomisation received $100. All participants engaged in those community venues received a packet including educational brochures about OSA, produced by the National Heart Lung and Blood Institute and the National Sleep Foundation, as well as infographics about OSA produced by the investigative team.

### 2.3 Survey data collection

#### 2.3.1 Measures obtained at baseline and follow‐up assessment

Before random group assignment, participants provided baseline sociodemographic variables including age, health insurance, marital status, education, place of birth, household composition, self‐described ethnicity, and annual household income. They also provided medical data including history of sleep disorders, obesity, diabetes mellitus, hypertension, respiratory disease, stroke, and heart disease, as well as a history of smoking and drinking habits.

We used the Apnea Risk Evaluation System (ARES™) ARES questionnaire to identify individuals who were at risk of OSA because of its accuracy in evaluating populations with a large pretest OSA probability (Levendowski et al., 2007). In the past 10 years, we have used the ARES questionnaire to screen 1250 black patients in primary‐care settings and 1092 blacks in community settings with no documented refusals (Rogers et al., 2015; Rogers et al., 2020). The questionnaire has a sensitivity of 0.94, specificity of 0.79 (based on a clinical cut‐off of AHI >5), positive predictive value of 0.91, and negative predictive value of 0.86. We also asked participants in both arms to complete the Dysfunctional Beliefs and Attitudes about Sleep scale (DBAS) (Morin et al., 2007), the Beck Anxiety Inventory (BAI) (Beck et al., 1988), the Centre for Epidemiologic Studies Depression Scale (CESD) (Baca‐Garcia et al., 2001), the Apnea Knowledge Test (AKT) (Smith et al., 2004), the Apnea Belief Test (ABT) (Smith et al., 2004) the Medical Outcomes Study Short Form (McHorney et al., 1993), the Self‐Efficacy Scale (Shahid et al., 2011), and the Change Assessment Scale (CAS) (Dozois et al., 2004) during the baseline and follow‐up outcome ascertainment. Additionally, we used the Interpersonal Processes of Care questionnaire during the follow‐up assessment. This is a Likert rating scale capturing the degree to which educators were compassionate, provided social support, showed concern about their feelings, respected them, and treated them as equal (Stewart et al., 2007).

#### 2.3.2 Randomisation to intervention or attention‐control arms

Participants were randomly assigned to either the intervention arm (paired with a peer sleep health educator trained to dispense the culturally and linguistically tailored sleep health education) or the attention‐control group (paired with a peer health educator who only received training in healthy lifestyle recommendations). The data manager generated a block randomisation sequence to ensure equal allocation and distribution of baseline characteristics of eligible participants to study arms. Using the randomisation table, the study coordinator randomly assigned participants to either the treatment or the attention‐control groups. Except for the study coordinator and the data manager, all study staff were blinded as to participant assignment. Outcome ascertainment during the follow‐up interviews were performed by a study staff who was also blinded as to group assignment.

##### Intervention arm

Participants in the intervention arm received tailored sleep health information and social support from trained peer sleep health educators (see details below). Participants received 10 sessions with their assigned health educator, intended to motivate the participant along the OSA continuum of care, which entails home‐based OSA assessment, and clinic referrals for further assessment and treatment. They also received peer‐based social support for 6 months as they went through the process of obtaining sleep evaluations and cues to action to overcome decisional conflicts.

##### Attention‐controlled arm

Consistent with Behavior Change Consortium (BCC's) recommendations, participants in the attention‐controlled arm received standard sleep literature, providing information about OSA and access to available sleep services. They also received information about the importance of healthy lifestyle changes to improve overall health and information about available sleep services. Of note, peer health educators in the control arm did not receive training in dispensing tailored sleep health education to participants; they only received overall training in dispensing information about lifestyle factors and the importance of preventive health behaviour. However, as in the intervention arm, participants received 10 sessions where their assigned health educator discussed their general health issues; participants received social support while endeavouring to address their own concerns about sleep and progress toward receipt of recommended health care.

## 3 INTERVENTION

The peer‐delivered tailored OSA behavioural intervention was patterned after our published Tailored OSA Behavioural Health Model, initially developed to navigate blacks with OSA and metabolic syndrome in the primary‐care settings (Jean‐Louis et al., 2017). Basically, the model uses a stage‐based approach, as proposed in the Prochaska's Stage of Readiness model, to address the perceived benefits of OSA evaluation and treatment, social factors that encourage or discourage desired OSA evaluation and treatment behaviour, as well as known facilitating and inhibiting factors derived from engagement of black patients discussing their own OSA journey (Jean‐Louis et al., 2017). Once the training manual was successfully adapted to reflect specific needs of community‐dwelling blacks with OSA symptoms and strategies to dispense OSA education in community settings (Seixas et al., 2018), it was presented to our Community Steering Committee for final endorsement before commencing training of the peer health educators, lasting 8 weeks. The 15‐year experience of our investigative team conducting patient‐engaged research has demonstrated the inclusion of a Community Steering Committee – comprising community leaders and health champions (barbershops, beauty salons, places of worship) – is essential (Lindenmeyer et al., 2007). It enables identification or refinement of relevant research questions (Earp et al., 2002), delineates barriers, and increases community participation (Sung et al., 2003), improves effectiveness of interventions (Westfall et al., 2007), and enhances dissemination of results (Lindenmeyer et al., 2007).

The conceptual framework for training peer sleep health educators was modelled after BCC's best practices and recommendations (Resnick et al., 2005). Specifically, the training plan incorporated educational modules describing the basics of health intervention, utilisation of motivational enhancement techniques to lead behaviour change, appropriate use of readiness level for personalising message delivery, and messages regarding the importance of OSA care to prevent negative health outcomes. All OSA educational messages were obtained from our previous RCT (Jean‐Louis et al., 2017); they were phrased in English at a fifth‐grade reading level by a professional linguist who is familiar with the Flesch–Kincaid readability scoring system and underwent usability testing to ensure that they were culturally sensitive, linguistically appropriate, practical, feasible, and acceptable to black patients (Zuvekas et al., 1999). For blacks with low levels of motivation, the initial task of the educator was to increase motivation and commitment to receiving an OSA evaluation. For those who were already motivated, the educator facilitated purposive action toward evaluation goals. Motivation was enhanced by increasing knowledge, modifying beliefs, and by increasing the value placed on OSA care, according to the core principles of Motivational Enhancement (Rubak et al., 2005), grounded in the Social Learning Theory (Bandura, 1977), and the Transtheoretical Model of Change (Prochaska et al., 1997). Upon completion of training, peer health educators were certified using an evaluation scheme ascertaining the following competencies: (1) mastery of OSA and sleep health information, (2) Readiness to Change model, and (3) interpersonal skills (clear and effective communication, cultural sensitivity, ability to create positive and effective rapport). Quarterly booster sessions were held to reinforce treatment fidelity, to address any project‐related problems they may be encountering, and to foster self‐confidence, self‐efficacy, and enthusiasm for the study. A full description of the intervention components and procedures for training the health educator is published elsewhere (Seixas et al., 2018).

### 3.1 Primary outcome: Home‐based **OSA** evaluation

The primary outcome is defined as adherence to physician‐recommended home‐based evaluation post intervention exposure. Once a commitment was made to undergo home‐based recordings, participants received two home visits. During the first visit, they received instructions as to the proper utilisation of the WatchPAT™ portable sleep device and how to provide data using the sleep diaries. The WatchPAT (Itamar Medical, Tel Aviv) is an FDA‐cleared, CMS approved device to assess sleep apnea severity. The self‐applied device measures peripheral arterial volume changes using a finger‐mounted plethysmograph. Information is collated with pulse oximetry along with heart rate, analysed based on a validated automated algorithm. This algorithm integrates arousals with SAO_2_ measurement to determine respiratory effort‐related arousals, showing excellent sensitivity and specificity for identifying OSA, defined as an apnea–hypopnea index (AHI) with a 4% desaturation ≥5, contrasted with NPSG. Meta‐analysis shows the respiratory disturbance index emanated from WatchPAT and polysomnography had a combined correlation of 0.88 (95% CI: 0.85–0.90; *p* < 0.001), whereas those comparing AHI4% had an R of 0.89 (95% CI: 0.86–0.92; *p* < 0.001). Based on Medicare and AASM guidelines (American Academy of Sleep Medicine, 2022), blacks with an AHI ≥10 were referred for treatment. Of note, we chose this criterion to ensure that we reduced the rate of false‐negative results. During the second visit, the research assistants retrieved the WatchPAT for data processing at the office.

### 3.2 Secondary outcome: Psychosocial measures

Participants with an AHI (≥ 10) were referred to the NYU Sleep‐Disorders Clinic for a consultation with a sleep physician and to receive recommended OSA care. Those assigned to peer sleep health educators received additional information regarding the laboratory‐based procedures and further support as participants navigated this phase of their OSA care; they also received assistance with scheduling appointments as the need arose. The purpose of this was to demystify the OSA assessment (including sleep consultation and overnight polysomnographic recordings); blacks in previous research intimated that this was a significant barrier and often hindered their adherence to laboratory‐based assessment. Participants in the control arm were also contacted by their assigned health educators to assess the level of engagement of the OSA care process and to encourage them to follow physician‐recommended care, but they received no tailored OSA information.

At the end of the 12 month enrolment period, participants in both arms were debriefed by a study member of staff, who was initially blinded as to assignment to study arms. Participants completed the same psychosocial questionnaires administered at baseline and additional post‐intervention questionnaires designed to assess rapport and support received from the assigned educators. These included an adapted component of the Interpersonal Processes of Care (Emotional/Companionship). This captured Likert scale ratings of the degree to which educators were compassionate, provided social support, showed concern about their feelings, respected them, and treated them as equal (Stewart et al., 2007). Capturing these process data was necessary to enable us to understand why participants adhered (or failed to adhere) to the intervention and how the intervention might be refined to be more effective.

### 3.3 Statistical analysis

While the study was initially intended to be deployed as a cluster randomised trial, most of the participants were recruited from a large church in Brooklyn, NY. Thus, the analysis plan used a RCT design to assess the effectiveness of the intervention (Jean‐Louis et al., 2017). Of note, no significant differences were noted comparing individuals recruited from the church and other sites (barbershop, beauty salons, food pantries, and civic organisations). We performed analysis based on intent‐to‐treat assuming that all patients in the intervention arm were exposed to peer‐delivered tailored OSA health messages and social support. Before assessing intervention effects, we verified comparability of patients in the control and intervention arms with respect to all available baseline sociodemographic and clinical data. We then compared patients who adhered to recommended OSA care and those who did not post‐enrolment using multivariate adjusted logistic modelling. This permitted estimation of the adherence rates to recommended home‐based OSA evaluation and clinic‐based evaluation. Although the study was designed to assess adherence to recommended treatment, very little data were available to support meaningful analysis due to a large attrition rate. Adherence was a binary outcome (yes vs. no) for all the measured outcomes (home‐based OSA evaluation and clinic‐based evaluation). While the primary objective was to ascertain the effectiveness of the peer‐delivered OSA education, the secondary objective was to examine the factors that might have influenced adherence behaviour, if it occurred. Candidate predictors including sociodemographic factors, health risk, medical conditions, and psychosocial factors were examined. We also assessed the unique contribution of social support as a predictor of adherence behaviour, but this factor could not be randomised given the inherent difficulty of controlling the degree of peer‐based social support in behavioural interventions.

All pre‐planned analytic methods, including power assessments, were previously detailed in our trial methods paper (Seixas et al., 2018). Accordingly, *t*‐test and Chi‐square tests were performed to compare each outcome with the putative factors depending on the type (continuous or categorical) and distribution (normal or not normal) of each variable. Comparisons involving multiple *t*‐tests were verified using analysis of variance to adjust for potential redundancy due to collinearity. Logistic regression modelling was employed to assess the likelihood of adhering to recommended OSA evaluation (receipt of home‐based evaluation). Candidate psychosocial and medical variables were included in the multivariate‐adjusted logistic regression model. For each outcome, we provide point estimates along with a 95% confidence interval for all predictors in the models. To account for missing values in the psychosocial questionnaires, the average of each participant's completed questions was calculated. The average was then used or multiplied by the total number of questions (depending on the scoring method of the questionnaire). All statistical analyses were performed using the [R] software version 3.6.1 (R Core Team, n.d.).

## 4 RESULTS

Overall, descriptive analyses revealed that there were no significant differences in the sociodemographic characteristics of participants in the two study arms. Likewise, participants did not differ significantly regarding self‐reported health status and physician‐diagnosed chronic health conditions (Tables 1 and 2). We could not perform a robust comparison between baseline characteristics of volunteers from the recruitment sites to ascertain potential clustering effects, as most of them were enrolled from a large church in Brooklyn, NY. Nonetheless, preliminary comparisons of the descriptive characteristics of the participants indicated no differences.

**TABLE 1:** Values represent means/percentages for each measured baseline sociodemographic characteristic of volunteers in the attention‐controlled and intervention arms; group comparisons were performed using *t*‐ and Chi‐squared tests, showing no significant differences.

Variable	Control	Intervention	*p*
Age; Mean (SD)	53.0 (13.7)	50.2 (14.1)	0.078
Gender: Female (%)	60.8	63.9	0.642
Employment; Yes (%)	49.0	48.6	0.952
Marital status; Married (%)	31.1	25.9	0.393
Education			0.658
≤ High school (%)	43.9	46.1	
Some College/Assoc. degree (%)	31.8	27.0	
≥ Bachelor (%)	24.3	27.0	
Income			0.903
≤ $10,000 (%)	35.7	37.4	
$10,000–$39,999 (%)	31.5	29.0	
≥$40,000 (%)	32.9	33.6	
Alcohol history; Yes (%)	38.4	50.0	0.065
Smoking history; Yes (%)	41.3	47.8	0.406
Health insurance; Yes (%)	75.0	65.8	0.112
Body mass index			0.056
Underweight (%)	1.3	0.0	
Normal weight (%)	10.6	20.7	
Overweight (%)	30.5	28.0	
Obesity (%)	57.6	51.3	

**TABLE 2:** Values represent percentages for each measured baseline medical characteristic of volunteers in the attention‐controlled and intervention arms; group comparisons were performed using Chi‐squared tests, showing no significant differences.

Variable	Control	Intervention	*p*
Hypertension (%)	53.8	46.2	0.216
Heart disease (%)	12.0	11.4	1.000
Diabetes (%)	35.4	36.1	1.000
Stroke (%)	5.7	5.8	1.000
Depression (%)	33.5	27.8	0.329
Sleep apnea (%)	18.4	19.6	0.886
Respiratory disease (%)	5.1	4.4	0.980
Insomnia (%)	22.3	23.4	0.918
Anxiety (%)	48.7	45.2	0.607
Sleepiness (%)	60.5	57.4	0.660

In Table 3, we provide results of our *t*‐ and Chi‐squared tests comparing data on self‐reported measures for participants in the intervention and attention‐controlled arms, captured during the baseline ascertainment period. These results showed no significant differences in the baseline psychosocial outcomes, comparing the two study arms. Likewise, we observed no significant difference in the level of social support received from health educators in both arms.

**TABLE 3:** Values represent the average for each measured baseline psychosocial characteristic of participants in the attention‐controlled and intervention arms. Group comparisons were performed using Chi‐squared and *t*‐tests, showing no significant differences. Scales included complete the Dysfunctional Beliefs and Attitudes about Sleep scale (DBAS), the Center for Epidemiologic Studies Depression Scale (CESD), the Beck Anxiety Inventory (BAI), the Apnea Knowledge Test (AKT), the Apnea Belief Test (ABT), and the Change Assessment Scale (CAS).

Variable	Control	Intervention	*p*
Risk perception (mean ± SD)	18.59 (4.86)	18.02 (5.39)	0.373
Outcome expectancy (mean ± SD)	27.07 (7.65)	26.33 (7.75)	0.447
Treatment self‐efficacy (mean ± SD)	23.34 (7.86)	22.77 (8.31)	0.569
DBAS (mean ± SD)	5.64 (2.02)	6.00 (1.72)	0.128
AKT (mean ± SD)	6.62 (2.13)	6.22 (2.48)	0.165
ABS (mean ± SD)	76.71 (9.75)	77.80 (7.84)	0.291
CESD (mean ± SD)	18.29 (12.14)	18.81 (12.33)	0.720
BAI (mean ± SD)	17.98 (14.58)	15.62 (12.36)	0.165
CAS (mean ± SD)	8.81 (2.08)	8.90 (1.78)	0.689

The results of our Chi‐squared tests indicated no significant differences comparing the adherence rates for recommended home‐based OSA evaluation between the intervention and the control arms (45.9% vs. 45.6%, NS). Similarly, the two study arms did not differ significantly with respect to the adherence rates for recommended laboratory‐based evaluation (48.8% vs. 46.5%, NS). As shown in the flow chart, we did not capture sufficient data to permit a meaningful comparison between the number of volunteers adhering or not adhering to recommended OSA treatment.

While the exposure to tailored sleep health education did not seem to translate preferentially into greater adherence rates, relative to exposure to standard OSA literature and healthy lifestyle recommendations, we noted that individuals in both arms agreeing to undergo OSA evaluation were likely to have experienced a greater level of peer‐based social support compared with those who declined referrals for an evaluation (8.23 ± 2.36 vs. 7.31 ± 2.35; *p* = 0.06). Furthermore, we observed those individuals adhering to such recommendations exhibited significantly greater DBAS and ABS scores than those who declined them (6.0 ± 1.8 vs. 4.9 ± 2.2; *p* = 0.02 and 77.0 ± 7.1 vs. 73.2 ± 7.4; *p* = 0.04, respectively); AKT was also higher among adherers, relative to non‐adherers (6.44 ± 3.1 vs. 7.6 ± 2.4; *p* = 0.06). However, other psychosocial measures did not show significant differences between patients receiving OSA evaluations versus those who did not (Table 4).

**TABLE 4:** Values represent the psychosocial characteristics of all participants who underwent a home‐based OSA assessment vs those who did not at follow‐up. Group comparisons were performed using *t*‐tests; MANOVA results were nearly identical with *t*‐tests. Scales included the Dysfunctional Beliefs and Attitudes about Sleep scale (DBAS), the Center for Epidemiologic Studies Depression Scale (CESD), the Beck Anxiety Inventory (BAI), the Apnea Knowledge Test (AKT), the Apnea Belief Test (ABT), and the Change Assessment Scale (CAS).

Variable	Home study: No	Home study: Yes	*p*
Risk perception (mean ± SD)	17.21 (5.69)	19.11 (5.52)	0.151
Outcome expectancy (mean ± SD)	8.00 (2.54)	26.33 (7.75)	0.564
Treatment self‐efficacy (mean ± SD)	21.91 (8.41)	24.29 (8.06)	0.236
CAS (mean ± SD)	8.26 (2.41)	8.71 (1.73)	0.338
DBAS (mean ± SD)	4.93 (2.20)	6.00 (1.79)	**0.024**
ABS (mean ± SD)	73.24 (7.44)	76.98 (7.11)	**0.041**
AKT (mean ± SD)	6.44 (3.03)	7.58 (2.41)	0.061
CESD (mean ± SD)	17.62 (12.40)	16.91 (13.12)	0.827
BAI (mean ± SD)	16.86 (14.83)	18.01 (13.40)	0.737
Social Support (mean ± SD)	7.34 (2.41)	8.26 (2.38)	0.063

We also compared the average change scores (12‐month minus baseline measures) between the adherers and non‐adherers, finding that the CESD change score showed a significant improvement among adherers, relative to non‐adherers (mean difference: −2.6 ± 9.7 vs. 2.9 ± 8.5, *p* = 0.02); no appreciable differences in baseline and follow‐up measures were noted for the other psychosocial variables (Table 5).

**TABLE 5:** Values represent the average change scores (baseline – follow‐up assessments) for the psychosocial characteristics of participants who underwent an OSA evaluation vs those who did not. Group comparisons were performed using *t*‐tests; MANOVA results were nearly identical with *t*‐tests. Scales included the Dysfunctional Beliefs and Attitudes about Sleep scale (DBAS), the Center for Epidemiologic Studies Depression Scale (CESD), the Beck Anxiety Inventory (BAI), the Apnea Knowledge Test (AKT), the Apnea Belief Test (ABT), and the Change Assessment Scale (CAS).

Variable	Home study: No	Home study: Yes	*p*
Risk perception (mean ± SD)	−1.04 (4.56)	0.93 (5.30)	0.110
Outcome expectancy (mean ± SD)	−1.05 (9.13)	−0.18 (8.85)	0.703
Treatment self‐efficacy (mean ± SD)	−0.95 (7.39)	1.07 (8.47)	0.318
DBAS (mean ± SD)	−0.58 (1.79)	−0.29 (1.47)	0.458
ABS (mean ± SD)	−3.10 (10.32)	−0.40 (8.33)	0.228
AKT (mean ± SD)	0.28 (3.18)	1.00 (2.80)	0.291
BAI (mean ± SD)	1.81 (14.81)	0.12 (13.33)	0.622
CESD (mean ± SD)	2.08 (9.07)	−2.62 (9.74)	**0.029**
CAS (mean ± SD)	−0.53 (1.98)	−0.19 (1.94)	0.478

As illustrated in Table 6, the results of the adjusted logistic regression modelling the association of sociodemographic and medical factors with adherence to recommended OSA evaluation showed that DBAS was the strongest predictor of adherence status (OR = 1.35; 95% CI: 1.03–1.81). ABS was no longer a significant predictor when both DBAS and ABS were entered in the model simultaneously. When the demographic cluster (age, sex, education, and income) was entered in the model, DBAS was no longer a significant predictor (OR = 1.31 95% CI: 0.95–1.86). Likewise, when the medical cluster (obesity, respiratory disease, and daytime sleepiness) was entered in the model, DBAS was no longer a significant predictor (OR = 1.30; 95% CI: 0.97–1.76).

**TABLE 6:** Results of the adjusted logistic regression analysis, modelling the association of baseline sociodemographic and medical factors with adherence to recommended home‐based OSA evaluation (model 1). In model 2, we considered the contribution of sociodemographic factors, and in model 3, the contribution of medical factors. Scales included only the Dysfunctional Beliefs and Attitudes about Sleep scale (DBAS) and the Apnea Belief Test (ABT), as they showed significant effects in preliminary analyses (Table 4).

	Model 1		Model 2		Model 3	
	OR (95% CI)	*p*	OR (95% CI)	*p*	OR (95% CI)	*p*
DBAS	1.35 (1.03–1.81)	**0.035**	1.31 (0.95–1.86)	0.110	1.30 (0.97–1.76)	0.086
ABS	1.00 (0.94–1.05)	0.949	0.98 (0.92–1.04)	0.586	1.00 (0.95–1.06)	0.924
Age	‐		0.97 (0.92–1.02)	0.203	‐	
Sex	‐		1.93 (0.55–7.09)	0.306	‐	
Education	‐		0.79 (0.23–2.62)	0.707	‐	
Income	‐		1.57 (0.47–5.39)	0.462	‐	
Sleepiness	‐		‐		1.08 (0.38–2.99)	0.882
Respiratory disease	‐		‐		0.49 (0.02–14.59)	0.637
Obesity	‐		‐		0.93 (0.34–2.53)	0.884

## 5 DISCUSSION

In this study, we used a stakeholder‐engaged approach to evaluate the effectiveness of peer‐delivered tailored OSA health education (PEERS‐ED) and of social support in increasing adherence to recommended OSA evaluation among blacks in community settings (e.g., barbershops, beauty salons, places of worship) throughout New York City. Our analyses yielded two important observations. First, the results suggested no significant differences in the adherence rates of recommended OSA evaluation between individuals in the intervention and the control arms, whether we considered home‐ or laboratory‐based settings. Second, whereas exposure to PEERS‐ED did not engender greater adherence rates, relative to exposure to standard OSA and healthy lifestyle information, individuals who underwent OSA evaluation may have experienced greater level of social support from health educators and were characterised by significantly greater DBAS and Apnea Beliefs Scale (ABS) scores and reduced depressed moods than those declining such recommendations.

The central hypothesis of this trial is that exposure to PEERS‐ED would enable blacks at risk of OSA to navigate successfully through the complex process of making autonomous decisions regarding OSA evaluations (Seixas et al., 2018). The finding of no appreciable difference between the two study arms was unexpected, given the positive intervention responses we observed in two previous clinical trials using a similar patient‐centred approach. In both RCTs, exposure to tailored OSA materials among blacks led to improved behavioural outcomes. Specifically, the first study testing the effectiveness of telephone‐delivered tailored OSA education showed that blacks in the intervention arm were 3.17 times as likely as those in the attention‐controlled arm, receiving standard OSA literature, to adhere to recommended OSA care (Jean‐Louis et al., 2017). In the second study, we observed that blacks exposed to tailored OSA content, dispensed via web‐based video vignettes featuring black patients describing their OSA journey, showed a significantly greater level of OSA self‐efficacy relative to those exposed to standard online OSA materials (Jean‐Louis et al., 2020). Overall, this finding suggested greater likelihood of adherence to recommended OSA care, as self‐efficacy is a strong predictor of adherence status. We note, however, that we could not establish whether blacks showing high self‐efficacy in effect adhered to recommended OSA care, as a direct assessment of that behaviour, as it was beyond the scope of that study.

Whereas the delivery of health education is generally associated with enhanced adherence to recommended medical care, there are instances where such interventions do not yield the desired behavioural change outcomes, although we note few of those interventions were aimed at improving adherence to OSA recommendations (Cook, 2008). Despite mixed findings concerning the effectiveness of such approaches, the present finding offers inconclusive evidence that tailored OSA education had absolutely no influence on individual behaviour leading to adherence to OSA evaluation. Indeed, our debriefing interviews with individuals in both arms suggested that those exposed to tailored OSA education would have preferentially undergone OSA evaluation, but it is unclear whether recall bias may have affected those subjective reports. Additionally, we observed no differences in the psychosocial measures that could explain this discordant finding. In effect, self‐efficacy scores, the best indicators of adherence behaviour, showed no significant difference between the two study arms at baseline or during the follow‐up assessments.

While the main objective of this trial was to assess the effectiveness of PEERS‐ED, the present findings favoured an important role of peer‐based social support in delineating the likelihood of increased adherence to recommended care. Social support in this context is defined as the subjective experience of care, affection, value, belonging, or assistance of various social networks (Stewart et al., 2007). We observed that individuals, reporting a greater level of peer‐based social support, were more likely to adhere to OSA evaluations, whether they were exposed to tailored OSA education or not. This finding bordered significance, which could be explained by the relatively large attrition rate, limiting our statistical power. Notwithstanding this limitation, it is highly suggestive that peer‐based social support is a key ingredient of behavioural change (Taylor & Seeman, 1999). Indeed, our observation is consistent with a recent report indicating support received from peers with OSA led to increased adherence to OSA care (Parthasarathy et al., 2013). Additionally, previous studies we conducted among blacks in minoritised communities revealed that blacks receiving social support from family and/or friends had a greater ability to cope with daily challenges and health outcomes (Jean‐Louis et al., 2007; Seixas et al., 2018). Others have reported that a higher level of social support is generally associated with greater adherence to medical care among patients with chronic health conditions and sleep outcomes (Kent de Grey et al., 2018). It is of interest to assess the synergistic effect of OSA education and social support on adherence behaviour, which would require that individuals be assigned to study arms providing OSA education with or without social support versus those receiving standard care.

Consistent with published reports suggesting psychosocial factors are generally associated with adherence behaviour, we found that individuals who underwent OSA evaluation exhibited significantly greater DBAS and ABS scores than those declining such recommendations. It is interesting that the two scales capturing beliefs about sleep both showed improvement over time. This suggests that the mere participation in the study might have activated blacks with OSA to adjust their maladaptive beliefs about sleep, thus reducing decisional conflicts and misgivings about adhering to recommended OSA evaluations. This level of activation might have also engendered an increase in knowledge about OSA care and could explain the reduced depression scores among adherers, which is not surprising as meta‐analyses revealed depression is often associated with poor adherence to medical care (Zuvekas et al., 1999). However, other psychosocial measures (e.g., anxiety level, self‐efficacy, and change assessment score) did not show significant differences between participants receiving OSA evaluations and those who did not. While many of those measures may not have yielded consistent results across studies (Zuvekas et al., 1999), self‐efficacy proved an independent contributor in our previous multivariate models predicting adherence status (Jean‐Louis et al., 2017). An important caveat is worth noting. Plausibly, the self‐efficacy measure we used may not be as robust as previous analyses might have suggested. This assertion is in part supported by our previous research showing that only the “treatment self‐efficacy” component was associated with a greater likelihood of seeking OSA care, whereas the other two components: outcome expectancy and risk perception were not significant predictors (Jean‐Louis et al., 2017).

Our analyses also revealed none of the sociodemographic or medical factors predicted OSA adherence status. It might have been expected that patients at risk for OSA would have been more inclined to seek OSA care since it involves the respiratory system and causes daily bouts of debilitating sleepiness (Rogers et al., 2015), which are more common among blacks. Likewise, other conditions such as obesity, diabetes, and hypertension, which are also more common among blacks (Beck et al., 1988), did not prove significant motivating factors anchoring positive actions toward adherence to OSA care. It is well established that patients with OSA experience clinically meaningful improvement in all those metabolic conditions (Sharma et al., 2011). A more focussed effort seems necessary to help alleviate the OSA burden among blacks, as they continually present with more severe OSA at the time of diagnosis and exhibit worse adherence profile compared with other racial/ethnic groupings, which might underlie suboptimal OSA‐related cardiovascular outcomes.

### 5.1 Limitations and strengths

An important limitation of our trial is that it is not generalisable to other racial/ethnic groups, since it was intended to address specific barriers elicited from direct engagement of blacks at risk for OSA. We observed a relatively higher than expected attrition rate (Figure 1), relative to rates observed in our previous research. This affected the follow‐up outcome ascertainment particularly regarding the determination of adherence to prescribed OSA treatment, albeit a secondary outcome. Furthermore, the use of intention to treat analysis precludes the need to assess systematic bias due to the attrition rate, although we found no significant differences in baseline characteristics contrasting completers and non‐completers. Beyond the factors that usually influence attrition rates, we note that relocation of our sleep clinic to a non‐NYU affiliated hospital was detrimental to our ability to retain participants in the trial because of uncertainties in scheduling clinic visits.

Another important limitation relates to the use of one component of the Interpersonal Processes of Care survey to capture peer‐based social support, which could not be randomised given the inherent challenge of controlling degrees of social support. Furthermore, while this was consistent with published studies, the use of a more robust scale might have provided stronger data to support definitive conclusions regarding the influence of social support on adherence behaviour. Utilisation of a multidimensional social support scale with established psychometrics for diverse samples incorporating items such as contact with others (i.e., bed partner, friends, and relatives) and belongingness to a place of worship or other informal groups is highly recommended (Williams et al., 2017). It bears noting that we cannot discount the possibility that interactions with study personnel could have contributed to individual self‐activation to follow through with recommended OSA care, beyond the health information they received from their health educators, although study personnel did not discriminate regarding the level of interactions with individuals in the study arms, as they were blinded to group assignment. We also note that the finding of no direct effects of the intervention on adherence status could not be explained by intervention drifts (Type III error), as all fidelity checks (e.g., adherence to training manual, booster sessions, review of intervention checklists) were performed as planned. Likewise, cross‐contamination could not explain these results based on information gathered from the debriefing interviews. Despite such limitations, the results of our study point to a significant role of psychosocial factors including social support in increased adherence to physician recommendation for OSA evaluation. Success of the peer‐based social support, mitigating the negative effect of socio‐cultural factors and decisional conflicts, may be in part attributable to its ability to reduce maladaptive beliefs about sleep. Our previous research revealed a high level of dysfunctional beliefs and attitudes about sleep among blacks (Williams et al., 2017).

## 6 CONCLUSIONS

Consistent with the national mandate to improve health equity among US racial/ethnic groups, there is an urgent need to implement novel behavioural models to navigate blacks with OSA symptoms in the healthcare system. While the delivery of health education is generally associated with enhanced adherence to recommended medical care, the results of the present study favoured an important role of peer‐based social support in the decision to adhere to recommended OSA care, above and beyond the potential effect of tailored OSA messages. Of interest is the finding that the two scales capturing beliefs about sleep, namely the Dysfunctional Belief About Sleep and the Apnea Beliefs scales, both showed improvement over time. This suggests that participation in the study itself might have been a catalyst to activate blacks in the process of seeking OSA care, likely through a reduction in maladaptive beliefs about sleep. Future studies should investigate the contribution of peer‐based social support using more robust social support measures or the synergistic effects of OSA education and social support on OSA adherence behaviour (Rogers et al., 2023; Watach et al., 2022).

## AUTHOR CONTRIBUTIONS

**A. Rogers:** Writing – original draft; writing – review and editing; methodology; data curation. **Girardin Jean‐Louis:** Conceptualization; investigation; writing – original draft; methodology; validation; writing – review and editing; funding acquisition; visualization. **P. Jin:** Writing – original draft; writing – review and editing; methodology. **R. Moise:** Writing – review and editing. **J. Blanc:** Writing – review and editing. **O. M. Bubu:** Writing – review and editing. **D. Chung:** Writing – review and editing. **F. Zizi:** Writing – review and editing. **A. A. Seixas:** Methodology; writing – review and editing.

## FUNDING INFORMATION

The authors report no conflicts of interest. This research was supported by funding from the National Institutes of Health: K07AG052685, R01HL142066, R01AG067523, R01AG056031, R01MD007716, K01HL135452, and R01HL152453. The funding sources had no role in the design, conduct, or analysis of the study, or in the decision to submit the manuscript for publication.

## CONFLICT OF INTEREST STATEMENT

The authors have no conflicts of interest to declare.

## Data Availability

The data that support the findings of this study are available from the corresponding author upon reasonable request.
